# Improvement of conjunctival cytological grade and tear production in Ankylosing Spondylitis patients under TNF inhibitors: a long-term follow-up

**DOI:** 10.1038/s41598-019-57266-1

**Published:** 2020-01-15

**Authors:** Fany Solange Usuba, Carla Gonçalves Schahin Saad, Nadia Emi Aikawa, Priscila Novaes, Julio Cesar Bertacini Moraes, Ruth Miyuki Santo, Jozelio Freire Carvalho, Eloisa Bonfá, Milton Ruiz Alves

**Affiliations:** 1 Ophthalmology Department, Hospital das Clinicas HCFMUSP, Faculdade de Medicina, Universidade de São Paulo, São Paulo, Brazil; 2 Rheumatology Division, Hospital das Clinicas HCFMUSP, Faculdade de Medicina, Universidade de São Paulo, São Paulo, Brazil

**Keywords:** Conjunctival diseases, Corneal diseases

## Abstract

Dry eye disease can compromise the patient’s quality of life. Few studies assessed the ocular surface (OS) in Ankylosing Spondylitis (AS) patients. This study aimed to evaluate the clinical and cytological findings of the OS in patients with AS, classify dry eye disease (DED) severity grade and conjunctival impression cytology (IC), and the effects of TNF inhibitors (TNFi) in a one-year follow-up. A baseline (BL) evaluation included 36 AS patients and 39 healthy controls. They fulfilled the Ocular Surface Index Disease questionnaire and underwent the Schirmer I test, break-up time, vital staining, and conjunctival IC. A DED severity grade, as well as IC rating, was applied. Fourteen of these patients received TNFi and analysis of ocular and systemic AS disease parameters occurred at BL and three months (3 M), and 12 months (12 M) after treatment. The AS patients presented a higher frequency of DED (p = 0.01), a worse score of severity (p = 0.001), and a higher frequency of altered IC (p = 0.007) when compared to controls. The 14 patients under TNFi presented an improvement in all the clinical disease activity parameters throughout the one-year treatment (p < 0.05) even as a concomitant increase in the Schirmer test (p = 0.04), and a significant amelioration in the altered IC to a normal IC (p = 0.006). DED is a frequent and under-diagnosed ocular disease in AS patients. The long-term parallel improvement of disease activity and OS parameters in AS patients receiving TNFi suggests that the OS can be an additional target of systemic inflammation in AS.

## Introduction

Ankylosing spondylitis (AS) is a chronic inflammatory rheumatic disease associated with HLA-B27, axial involvement, and the presence of extra-articular manifestations such as involvement of the eyes, skin, and gastrointestinal tract^1^. Anterior uveitis is the classical ocular manifestation in AS. However, keratoconjunctivitis sicca (KCS), generally known as dry eye disease (DED), could also occur^2–4^. There are few and inconsistent reports regarding the simultaneous occurrence of AS and DED, even though the eye dry eye tests performed in these studies were incomplete^5–7^.

The clinical expression and pathophysiology of DED have recently changed^8,9^. DED is a multifactorial disease of the ocular surface (OS) characterized by a loss of homeostasis of the tear film accompanied by symptoms of ocular discomfort, visual disturbance, and tear film instability with potential harm to the ocular surface and visual function^10^. There is instability, hyperosmolarity of the tear film, neurosensory abnormalities, and inflammation of the OS, leading to damage of the surface epithelial cells, with the release of proinflammatory cytokines, chemokines, and matrix metalloproteinases in the tear film and the OS tissue^10,11^. Inflammation and apoptosis of OS cells might play a role in the development of DED, promoting loss of goblet cells and a decrease of mucin secretion^12^. The conjunctival impression cytology from DED patients comprised these alterations^13^. An experimental study demonstrated that TNF-α and IFN-γ induced apoptosis of goblet cells^14^.

Recently, the use of biologic agents has been investigated for the treatment of inflammatory eye diseases^15^, aiming at a more accurate target and with the potential for preventing disease progression^15,16^. Proinflammatory cytokines, such as TNF-α, play a role in the multifactorial cyclical mechanistic scheme of DED^11^. Few studies have evaluated the effect of TNF inhibitors (TNFi) therapy on the OS, and there are no data regarding AS^17–22^.

The objective of this study was to evaluate the frequency and severity of DED in AS patients using a complete systematic evaluation of the OS, including the cytological grade. Moreover, this study analyzed the longitudinal effects of TNFi on OS parameters.

## Methods

### Subjects

This prospective, observational study included 36 consecutive patients over 18 years of age with AS defined by the New York criteria^23^ and 39 healthy volunteer controls matched for age and gender. Both groups were undiagnosed for DED. Clinical and laboratory features were obtained from the ongoing electronic database. The exclusion criteria were the use of medications that might induce DED, topical anti-inflammatory drugs, active ocular diseases, previous ocular surgical procedures, and the use of contact lenses. This study occurred at the Rheumatology Division and the Department of Ophthalmology of the Hospital das Clinicas HCFMUSP, Faculdade de Medicina, Universidade de Sao Paulo, Sao Paulo, BR.

From the initial 36 patients, 14 consecutive AS patients, refractory to conventional treatment with non-steroidal anti-inflammatory drugs (NSAIDs), and to disease-modifying anti-rheumatic drugs (DMARD) and who were eligible to receive TNFi therapy, were enrolled for the longitudinal analysis. Eligibility criteria for anti-TNF use followed the ASAS/EULAR recommendations^24^. The prescription of NSAID and DMARD was stable during the whole study. A prospective evaluation of the ophthalmological parameters, clinical and laboratory assessments occurred at baseline (BL) and after three and 12 months (3 M and 12 M, respectively) of biologic therapy. Those patients who presented mild or moderate KCS were oriented to use lubricant eye-drop four times per day but not on the day of evaluation.

All the clinical procedures followed the tenets of the Declaration of Helsinki. The Local Research Ethics Committee (Comissão de Ética para Análise de Projetos de Pesquisa – CAPPesq, HCFMUSP, CAAE 37166914.8.0000.0068) approved the study, and all the subjects signed informed consent.

### Ocular clinical measurements

At the baseline (BL) visit, a biomicroscopy examination performed under the slit lamp assessed the OS. A feature of eyelids, cornea, conjunctiva, and tear film was evaluated and recorded. All the patients underwent the Schirmer I test without topical anesthesia: the strips remained at the temporal third of the inferior eyelid of each eyelid of both eyes for 5 minutes. Afterwards, the wet extension of the strips according to the scale (0–35 mm) gave the value of the Schirmer test. Results below 10 mm were considered altered^25^.

Fluorescein strips (fluorescein 1,0 mg/mL) measured tear break-up time (BUT) wetted with 0.9% sodium chloride and applied to the inferior fornix. Values of 10 seconds or below were considered abnormal^26^.

Corneal and conjunctival vital staining: vital staining with fluorescein and lissamine green utilized impregnated strips (1,0 mg/mL for the fluorescein strips and 1,5 mg/mL for the lissamine green strips) wetted with 0,9% sodium chloride and applied to the inferior fornix. Initially, the instillation of fluorescein drop evaluated the graduation of the corneal staining pattern, followed by lissamine green conjunctival staining^26^. The score ranged from 0 to 9: values > 3 were considered abnormal^26^.

### Subjective ocular symptoms of dry eye

All subjects answered a self-reported questionnaire - the Ocular Surface Disease Index (OSDI), culturally adapted and validated for Brazilian-Portuguese^27^. This survey evaluated dry eye symptoms, their impact on daily life activities, and environmental triggers. The score (0–100) discriminated among normal (0–12), mild (13–22) to moderate (23–32), and severe (above 33) DED^28^.

### Dry eye severity grade

From the measurements described above, the DED severity grading scheme, according to the Behrens *et al*. modified classification method was applied^29^. The symptoms and the value of the clinical signs resulted in a disease severity grading score. The status of severity ranged from 1 to 4^29^.

Level 1 (mild): mild symptoms, no conjunctival or corneal staining, meibomian gland dysfunction (MGD) variably present, variable values of BUT (in seconds), and Schirmer (mm/5 min). Level 2 (moderate)- moderate symptoms, a variable score of conjunctival and corneal staining, MGD variably present, BUT ≤ 10 sec, Schirmer ≤ 10 mm/5 min. Level 3 (moderate/severe)- severe or frequent symptoms, moderate to marked conjunctival and corneal staining, MGD frequent, BUT ≤ 5 sec, Schirmer ≤ 5 mm/5 min. Level 4 (severe)- severe symptoms, severe conjunctival and corneal staining, MGD such as trichiasis, keratinization and symblepharon present, immediate BUT, Schirmer ≤ 2 mm/5 min. A masked observer assigned the severity grade system.

Patients presenting any level of DED severity were considered positive for overall DED.

All the ocular clinical measurements were performed within the same time of day (from 1:00 PM to 4:00 PM) at the same conditions of temperature and relative air humidity.

### Conjunctival impression cytology

Impression cytology (IC) collected samples of tissue from the temporal bulbar conjunctiva. Application of cellulose acetate filters (Millipore GVWP, 0.22-μm pore; Millipore Corp., Bedford, MA, USA) in this area was under local anesthesia with 0.5% proparacaine hydrochloride. The specimens were fixed in absolute ethyl alcohol and stained with periodic acid-Schiff (PAS)-hematoxylin stain. The samples were evaluated under light microscopy and graded into four stages according to Nelson’s grading system based on the epithelial cell morphology and the nucleus to cytoplasmic ratio, goblet cell density, and goblet cell cytoplasm^13^ (Table 1). Grade 0 was considered normal, and grades 1, 2 and 3, altered. Ten fields of each specimen were analyzed, and the most prevalent classification was the final grade^13,30^. The evaluation of all the samples succeeded under a microscope at the same magnification (400x) and in a masked assessment^30^.

**Table 1:** Conjunctival cytological grade according to Nelson’s grading system.

Findings	Grade 0	Grade 1	Grade 2	Grade 3
Cell size	Smaller (<1.0 μm^2^)	Small (1.0 μm^2^)	Large (>1.0 μm^2^)	Large (>1.0 μm^2^)
Cytoplasm	eosinophil	eosinophil	variable	basophil
Nucleus	large	small	small	picnotic/absent
Nucleus:cytoplasm ratio	1:2	1:3	1:4–1:5	1:6
Goblet cells	>500	350–500	100–350	<100
Goblet cells cytoplasm	PAS +++	PAS +++	PAS ++	PAS −

PAS- periodic acid-Schiff reagent.

### AS disease parameters

The assessments of disease activity and functional parameters in the 14 AS patients receiving anti-TNF treatment complied with the Bath Ankylosing Spondylitis Disease Activity Index (BASDAI), the Bath Ankylosing Spondylitis Functional Index (BASFI). Inflammation was evaluated using the erythrocyte sedimentation rate (ESR) by the modified Westergren method and the C-reactive protein (CRP) by nephelometry.

### Statistical analysis

Statistical analysis of the data was conducted using IBM SPSS (Statistical Package for Social Sciences), version 23.0. The results are shown as the mean and standard deviation or median (range) for the continuous variables and as the frequency (%) for the categorical variables. The comparisons were performed using Student’s t-test, Mann-Whitney test, and Friedman nonparametric test. The McNemar test and Wilcoxon rank-sum test adjusted by Bonferroni’s correction, and Spearman rank-order correlation was used as well. The statistical significance was set at p-value < 0.05.

### Ethical standard

All the clinical procedures followed the tenets of the Declaration of Helsinki. The Local Research Ethics Committee approved the study, and all the subjects signed informed consent.

## Results

### Demographic profile

The median age of the AS patients was comparable to that of the healthy controls (37.5 vs. 42.0 years, p = 0.08), as was the frequency of male gender (88.9 vs. 89.7%, p = 0.9). The mean disease duration was 17.8±11.8 years (Table 2). Nine (25.0%) patients had a previous diagnosis of anterior uveitis, and none presented active uveitis throughout the study.

**Table 2:** Demographic data, ocular surface and disease parameters of ankylosing spondylitis (AS) patients and healthy controls.

	AS (n = 36)	Healthy controls (n = 39)	p
**Demographic data**			
Current age (years)	37.5 (17–63)	42.0 (23–59)	0.08
Male gender (%)	88.9	89.7	0.95
**Ocular surface parameters**			
Schirmer’s test (mm/5 min)	18.0 (2–35)	15.0 (4–35)	0.94
Schirmer ≤10 mm/5 min (%)	38.8	25.6	0.32
Schirmer ≤5 mm/5 min (%)	19.4	10.3	0.34
BUT (sec)	8 (3–15)	7 (3–20)	0.67
Fluorescein stain (score)	0 (0–1)	0 (0–0)	0.30
Lissamine green stain (score)	0 (0–3)	1 (0–3)	**0.006**
OSDI (score)	6.2 (0–84.8)	2.5 (0–20.8)	0.13
Impression cytology (score)	1 (0–2)	0 (0–1)	**0.015**
IC altered (%)	55.5	12.8	**0.007**
MGD (%)	50	53.8	0.68
Overall dry eye (%)	80.5	43.6	**0.011**
**Severity of dry eye (%)**			
Mild	50.0	35.8	
Mild/moderate	22.2	7,8	0.003
Moderate	8.3	0	
**Disease parameters**			
ESR (mm/1^st^h)	15 (2–75)	2 (1–79)	**<0.001**
CPR (mg/L)	20.5 (0.3–81.9)	2.6 (0.1–5.9)	**<0.001**

BUT: break-up time; OSDI: ocular surface disease index; IC: impression cytology(altered: scores 1,2 and 3); MGD: meibomian gland dysfunction; ESR: erythrocyte sedimentation rate; CRP: C-reactive protein; p < 0.05.

### Ocular symptoms and clinical measurements

Symptoms of the dry eye measured by the OSDI score was similar in patients and controls [6.2 (0–84.8) vs. 2.5 (0–20.8), p = 0.1].

At baseline, no difference in objective DED tests evaluation was observed comparing patients and controls regarding Schirmer I score ≤ 10 mm/5 min [14 (38.8%) vs. 10 (25.6%), p = 0.3]; Schirmer score ≤ 5 mm/5 min [7 (19.4%) vs. 4 (10.3%), p = 0.3]; BUT ≤ 10 seconds (77.6% vs. 74.3%, p = 0.6), BUT ≤ 5 seconds (38.8% vs. 38.4%, p = 0.8) with similar median BUT [8 (3–15) seconds vs. 7 (3–20) seconds, p = 0.6) (Table 2). The median of lissamine green staining score was lower in the AS patients than in the controls [0 (0–3) vs. 1 (0–3), p = 0.006]; however, in both groups, the median scores were within the normal levels. Meibomian gland dysfunction (MGD) was present in 18 (50.0%) patients and 21 (53.8%) controls (p = 0.6).

### Dry eye severity grade and impression cytology

Concerning dry eye severity grade, there was a higher frequency (p = 0.003), a worse score [1 (0–3) vs. 0 (0–2), p = 0.001] in AS patients when compared to controls. A higher IC score [1 (0–1) vs. 0 (0–2), p = 0.01] was demonstrated in AS patients (Table 2). The IC was altered in 20 (55.5%) patients, as follows: 17 (47.2%) presented with grade 1, and three (8.3%) with grade 2; the controls exhibited with altered impression cytology (grade 1) in 12.8% of the cases (p = 0.007).

### Inflammatory disease parameters

Additionally, systemic inflammation was higher in AS patients who showed higher acute phase reactants than controls (ESR and CRP, p < 0.001) (Table 2).

### Prospective evaluation of AS patients under TNFi therapy

Analysis of co-medication in 14 AS patients under TNFi therapy revealed: four (28.5%) with prednisone, six (43%) with sulfasalazine; three (21%) with methotrexate; and one (7%) with leflunomide.

There was a significant improvement in all the disease and inflammatory parameters in the 14 patients receiving TNF-i therapy from BL vs. 3 M vs. 12 M (p < 0.05) (Table 3).

**Table 3:** Prospective analysis of ankylosing spondylitis patients before and during anti-TNF therapy (n = 14).

	BL	3M	12M	p
**Ophthalmological parameters**				
Schirmer’s test (mm/5 min)	10 (2–35)	17.5 (4–35)	20 (4–30)	0.04*
Schirmer ≤ 10 mm/5 min (%)	28.6	28.6	28.6	1.0
Schirmer ≤ 5 mm/5 min (%)	21.4	14.3	21.4	0.48
BUT (seconds)	7 (3–15)	8 (0–15)	8 (3–15)	0.87
Fluorescein staining (score)	0 (0–1)	0 (0–1)	0 (0–1)	0.81
Lissamine green staining (score)	0 (0–1)	0 (0–0)	0 (0–1)	0.36
OSDI (score)	0 (0–84)	0 (0–81)	4.1 (0–84)	0.64
Overall frequency of dry eye (%)	78,5	64,2	50	0.1
Dry eye severity (score)	1 (0–2)	0 (0–1)	0.5 (0–1)	0.005*^†^
IC (score)	1 (0–2)	1 (0–1)	0 (0–1)	**0.006**^**†‡**^
IC altered (%)	78.6	57.1	35.7	**0.03**^**†**^
**Disease parameters**				
BASDAI	5.1 (1.04–8.16)	2.1 (0–7.76)	3.0 (0–7.33)	0.009*^†^
BASFI	5.2 (1.31–8.49)	2.3 (0–8.25)	3.0 (0–7.1)	0.01*^†^
ESR (mm/1^st^h)	20 (7–67)	4 (2–23)	7 (1–35)	0.01*
CRP (mg/L)	29.6 (2.5–81.9)	2.73 (0.15–30.4)	3.02 (0.3–22.8)	<0.001*^†^

BL: baseline; 3 M: 3 months; 12 M: 12 months; BUT: break-up time; IC: impression cytology (altered: scores 1, 2 and 3); OSDI: Ocular Surface Disease Index; BASDAI: Bath Ankylosing Spondylitis Disease Activity Index; BASFI: Bath Ankylosing Spondylitis Functional Index; ESR: erythrocyte sedimentation rate; CRP: C-reactive protein.

*p < 0.05 between BL vs. 3 M ^†^p < 0.05 between BL vs. 12 M ^‡^p < 0.05 between 3 M vs. 12 M.

The median Schirmer score values in the AS patients receiving TNF-i drugs at baseline was 10 mm/5 min (2–35) with an increase at 3 M and 12 M [3 M: 17.5 (4–35) and 12 M: 20 (4–30) mm/5 min, p = 0.04]. There were no statistically significant differences in BUT values, in the fluorescein and lissamine green staining scores nor the OSDI score at BL, 3 M, and 12 M. An improvement of the altered IC (grades 1 and 2) to normal IC succeeded in the AS patients comparing BL and 12 M (78.6% vs. 35.7%, p = 0.03) as well as the IC score [BL: 1 (0–2) vs 3 M: 1 (0–1) vs 12 M: 0 (0–1), p = 0.006] and dry eye severity level score [BL: 1 (0–2) vs 3 M: 0 (0–1) vs 12 M: 0.5 (0–1), p = 0.005]. There were no significant correlations between the OSDI and the clinical DED measurements nor between the severity of dry eye and the AS disease parameters.

## Discussion

To our knowledge, this study is the first to demonstrate a high frequency of underdiagnosed mild to moderate DED in AS patients. Long-term evaluation of ocular surface changes in these patients revealed an improvement of Schirmer I, DED severity, and IC parameters after TNFi therapy.

The noteworthy contribution of this study is the use of systematic and validated dry eye clinical criteria. These assessments were reported to be useful in a systematic review and a recent multinational European Consensus Group recommendation^31,32^. We excluded factors strongly associated with DED, such as the use of antihistamines, tricyclic antidepressants, antihypertensive agents, and benzodiazepines^33^. Advanced age is a known cause of DED; consequently, only patients under 65 years old were included^34^. Additionally, equal and homogeneous gender distribution in patients and controls was relevant because hormone changes unbalance tear production and tear quality^35^. Therefore, after excluding these factors, AS may play a role in the development of DED, and the ocular surface and lacrimal gland may be target organs in the autoimmune process in AS.

We observed herein that the majority of AS patients, despite the lack of clinical complaints and objective tests for the dry eye without a usual pattern for DED, showed mild to moderate DED severity.

This classification comprises both aqueous deficient and evaporative components. The severity classification does not have a continuous linear relationship for mild to moderate DED. The range of values, when combined after following the severity grading system, results in different scores seen between cases and controls. Thus, in mild to moderate dry eye levels, a discreet variance of results expresses in different stages of severity. Once there is no gold standard for dry eye test to correlate with severity, this system allows early diagnosis, and the accuracy of severity classification, especially in mild cases of DED^32^.

The discordance between signs and symptoms of DED could be related to the mild DED level with compensatory mechanisms^34^, and the presence of MGD, which is more commonly asymptomatic^36^. The AS patients present a low score of symptoms by OSDI. Furthermore, recent studies revealed that less than 60% of subjects with objective evidence of DED are symptomatic^32,37^. So, a thorough evaluation of AS patients is necessary regardless of symptoms once the appreciation of symptoms alone will result in missing a significant percentage of DED patients^37^. Gunes *et al*. evaluated corneal thickness by Scheimpflug imaging and dry eye tests in 57 AS patients and concluded that thinner corneas could be affected by tear dysfunction and inflammatory processes^38^. Ortak *et al*. also found thinner corneas and lower dry eye tests in a group of 68 AS patients. Consequently, they suggested careful attention in surgical interventions such as photorefractive keratectomy and laser *in situ* keratomileuses in AS patients^39^. Despite the dysfunctional dry eye tests found in these studies, the authors did not determine the DED severity level.

In the present study, more than 50% of the patients had altered IC, and this finding indicated that the epithelial layers of the OS were affected by chronic inflammation leading to the reduction of goblet cells with consequent OS damage^14^. The significant elevation of the inflammatory disease parameters (ESR and CPR) in AS patients compared to the control group supports the role of inflammation in this process. Increased expression of adhesion molecules and inflammatory cytokines of the OS epithelium and the tear fluid was demonstrated in DED, reinforcing the importance of inflammation in the pathogenesis of this condition^11^. The conjunctival IC analyzes epithelial cells that are related to the conjunctival inflammatory and apoptotic pathways, goblet cells producing mucin MUC5AC, and playing defense and regulatory role on the homeostasis of the OS^14^. The analysis of conjunctival IC by Nelson’s grading score has four grades and evaluates the morphology of epithelial cells, nucleus/cytoplasm ratio, and the number of goblet cells^13^. Besides, this scoring system is well-validated^32^. The lack of goblet cells is associated with inflammatory disorders of the ocular surface and has a vital role in the mechanism of DED^29^. Therefore, despite the unusual DED diagnostic tests, AS patients present a higher frequency of altered IC and a higher score of IC indicating DED. Thus, the conjunctival IC can be used as an additional parameter for dry eye as well as analyze inflammation on the ocular surface. The ODISSEY European Consensus Group recently considered the IC as a determinant dry eye criteria for diagnosing the severity of DED as well as an objective marker for ocular surface damage^32^. The AS cohort presented mild to moderate IC score, almost similar to the DED severity grade. The score of altered IC is usually compatible with the severity of clinical disease^13^. The conjunctival IC is easy to collect, minimally invasive, and evaluates various ocular surface disorders such as allergy, ocular graft-versus-host disease, Sjögren’s syndrome^13,14^.

Artificial tears, the first-line therapy for DED, have no impact on inhibiting the activation of innate inflammatory pathways in response to a desiccating environment, nor on corneal/conjunctival staining and goblet cell density^40–42^. The severe dry eye might require systemic immunomodulatory therapy to inhibit the expression of pro-inflammatory cytokines^43^. There are only isolated case reports of anti-TNF treatment for DED, and the Dry Eye Workshop II (DEWS II) algorithm recently included this agent as a therapeutic option^43,44^.

The long-term evaluation of AS patients treated with TNFi revealed that the clinical/laboratory disease parameters improved concomitantly with the tear production, DED severity, and IC and DED scores. The increase in the aqueous tear production observed in the AS patients indicated that TNFi therapy might interfere with the OS metabolism and restore lacrimal gland acinar cells affected by proinflammatory cytokines^19,45^. Trousdale *et al*. studied the expression of the TNF inhibitor gene in the lacrimal gland of rabbits and found that this inhibitor gene promoted recovery of tear production and tear stability^18^. Li *et al*. investigated the effectiveness of topical infliximab in a mouse model of an experimental dry eye. They concluded that topical application of the drug could improve tear production and ocular surface irregularity, decrease inflammatory cytokines, and cells on the ocular surface and increase conjunctival goblet cell density^19^. Although the improvement in systemic inflammatory markers, disease activity, and IC scores observed in AS patients treated with TNFi, there was not a reduction in the vital staining score. This outcome is not different from a previous study that correlated vital dye staining scores and conjunctival squamous metaplasia measured by impression cytology^46^. These authors concluded that a correlation occurred in a subgroup of Sjögren Syndrome (SS) patients with aqueous deficiency DED but not in the other subgroups evaluated (non-SS aqueous deficiency DED, inflammatory meibomian gland dysfunction DED, atrophic meibomian gland dysfunction DED). Moreover, in primary SS, the DED parameters are more severe than secondary SS^25^.

Despite most AS patients present mild DED, the neglection of this condition cannot occur. DED induces a chronic disability, and it is a fundamental factor to consider when planning treatment intervention and to offer an improvement of the quality of AS patient’s life: DED can give a worsening in quality of life and chronic incapacity comparable to patients with angina^47^.

In conclusion, the prevalent underdiagnosed DED observed in the AS patients enrolled in this study and the long-term improvement of tear production and goblet cell density in those receiving TNFi therapy suggest that the OS can be an additional target of the systemic inflammation in this disease.

## Data Availability

The datasets generated and analyzed during the current study are available from the corresponding author on reasonable request.
